# Student’s *t*-Kernel-Based Maximum Correntropy Kalman Filter

**DOI:** 10.3390/s22041683

**Published:** 2022-02-21

**Authors:** Hongliang Huang, Hai Zhang

**Affiliations:** 1 School of Automation Science and Electrical Engineering, Beihang University, No. 37 Xueyuan Road, Haidian District, Beijing 100083, China; huanghl@buaa.edu.cn; 2 Science and Technology on Aircraft Control Laboratory, Beihang University, No. 37 Xueyuan Road, Haidian District, Beijing 100083, China

**Keywords:** Kalman filter, student’s *t* kernel function, maximum correntropy criterion, fixed-point iteration method, convergence analysis

## Abstract

The state estimation problem is ubiquitous in many fields, and the common state estimation method is the Kalman filter. However, the Kalman filter is based on the mean square error criterion, which can only capture the second-order statistics of the noise and is sensitive to large outliers. In many areas of engineering, the noise may be non-Gaussian and outliers may arise naturally. Therefore, the performance of the Kalman filter may deteriorate significantly in non-Gaussian noise environments. To improve the accuracy of the state estimation in this case, a novel filter named Student’s *t* kernel-based maximum correntropy Kalman filter is proposed in this paper. In addition, considering that the fixed-point iteration method is used to solve the optimal estimated state in the filtering algorithm, the convergence of the algorithm is also analyzed. Finally, comparative simulations are conducted and the results demonstrate that with the proper parameters of the kernel function, the proposed filter outperforms the other conventional filters, such as the Kalman filter, Huber-based filter, and maximum correntropy Kalman filter.

## 1. Introduction

The state estimation problem is ubiquitous in various applications, such as navigation [1], target tracking [2], and so on [3,4]. The common state estimation method is the Kalman filter (KF), which has been successfully used in many fields [5,6,7]. For the linear system with additive Gaussian noise, the KF can achieve optimal state estimation. But most systems in real world are usually nonlinear, which limits the application of KF. To solve the state estimation problem of the nonlinear system, many novel filters, such as the extended Kalman filter (EKF) [8], unscented Kalman filter (UKF) [9], quadrature Kalman filter (QKF) [10], cubature Kalman filter (CKF) [11], and so forth have been proposed in the last few decades. The basic idea of EKF is to linearize the nonlinear system by the Taylor expansion technique and truncate the Taylor series at the first-order term. As a result, EKF requires the system model to be differentiable and the Jacobian matrices need to be calculated, resulting in high computational complexity. Besides, for strong nonlinear systems, the first-order linearization will inevitably introduce non-negligible linearization errors, which may lead to the degradation of state estimate performance. The UKF utilizes the unscented transformation (UT) technique to achieve the nonlinear propagation of the mean and covariance of the system state, which avoids the derivation of the Jacobian matrices. Compared with EKF, it has better performance, especially for the strong nonlinear system. To further improve the estimation accuracy, various numerical integration methods are introduced into the filter, such as cubature rule-based filters and quadrature rule-based filters. These methods improve the numerical approximation accuracy of intractable integrals, which leads to more accurate characterization of original probability density functions, correspondingly resulting in an enhanced estimation accuracy [12].

Most of the above filters are based on the mean square error (MSE) criterion and can achieve satisfying state estimation accuracy when the system noises are Gaussian. However, the MSE can only capture the second-order statistics of the noise and is sensitive to large outliers. In many areas of engineering, such as the power system state estimation [13,14], maneuvering target estimation [15] and INS/GNSS integrated navigation [16], the noise may be non-Gaussian and outliers may arise naturally. As a result, the performance of the filters based on MSE may deteriorate significantly. To address these problems, many filters based on non-MSE criteria have been proposed. The Huber-based Kalman filter (HKF) [17] relies on Huber’s generalized maximum likelihood methodology which minimizes the combined minimum $l_{1}$ and $l_{2}$ norm. The residual is bounded by using Huber’s function, which suppresses the influence of outliers on state estimation and exhibits strong robustness with respect to deviations from the Gaussian noise. Maximum correntropy Kalman filter (MCKF) [18] is based on the maximum correntropy criterion (MCC) of information-theoretic learning (ITL) [19,20]. Since the correntropy can capture high-order statistics of the noise rather than the common second-order statistics, the state estimation accuracy of MCKF is better than that of KF in the presence of the non-Gaussian noise. To copy with more complicated non-Gaussian noises, minimum error entropy Kalman filter (MEEKF) [21] based on another important learning criterion, minimum error entropy criterion [22,23], was also proposed. The experiment results show the excellent performance of the MEEKF when the underlying system is disturbed by some complicated non-Gaussian noises. Later, various nonlinear filters based on these information-theoretic learning criteria were proposed, such as the maximum correntropy unscented filter [24], minimum error entropy unscented Kalman filter [25], and so forth [26,27,28].

The performance of the filters based on the information learning criterion heavily depends on the kernel function and its parameters. Currently, most of these filters are based on the Gaussian kernel function. However, in some real applications such as agile targets tracking [29], multipath estimation [30] and measurement outliers from unreliable sensors [31], the systems may be disturbed by heavy-tailed non-Gaussian noises. The Gaussian kernel function may not be the best choice. For the heavy-tailed noises, the Gaussian kernel-based filters may overlook the heavy-tailed properties, which leads to a decrease in the estimation accuracy. Therefore, a novel Cauchy kernel-based maximum correntropy Kalman filter (CKKF) was proposed in the Ref. [32]. Compared with MCKF in which the Gaussian kernel function is used, the performance of CKKF is better for multi-dimensional non-Gaussian noise. Meanwhile, the behavior of the CKKF is consistently stable when a different Cauchy kernel bandwidth is selected.

Recently, a new kernel called Student’s *t* kernel function was proposed based on Student’s *t* distribution and the Mercer theorem [33]. Compared with the Gaussian kernel function, Student’s *t* kernel function can better capture the heavy-tailed features of the noise. The experimental results showed the superiority of the Student’s *t* kernel function. Considering the advantages of Student’s *t* kernel function, Student’s *t* kernel function-based maximum correntropy criteria are applied to the KF algorithm, and a novel Student’s *t* kernel-based maximum correntropy Kalman filter algorithm (STKKF) is proposed in this paper. Compared with the Gaussian kernel function, Student’s *t* kernel function has two parameters, which can be used to control the shape and the kernel bandwidth of the kernel function, respectively. Thus, it can characterize the distribution of heavy-tailed noise more effectively. The comparative simulations show that with the proper parameters of the kernel function, the accuracy of state estimation of the STKKF outperforms that of conventional algorithms when the noises are heavy-tailed non-Gaussian.

The main contributions of this paper are summarized as follows:

- A novel maximum correntropy Kalman filter is developed in which the Student’s *t* kernel function is used to replace the conventional Gaussian kernel function.
- Considering the fixed-point iteration method is used to update the posterior estimates of the state in STKKF, the convergence analysis under a certain condition is given.
- The comparative simulations with other filters are conducted to demonstrate the superiority of STKKF.

The rest of the paper is organized as follows. In Section 2, basic knowledge about the correntropy and Kalman filter are introduced briefly. In Section 3, The STKKF based on Student’s *t* kernel maximum correntropy criterion is derived. The convergence of the filter is analyzed in Section 4. To evaluate the performance of STKKF, the comparative simulations are conducted in Section 5. Finally, a discussion is given in Section 6.

## 2. Preliminaries

### 2.1. Correntropy

The correntropy was proposed to measure similarity across lags as the autocorrelation of random processes [34], and then was extended to measure the localized similarity of arbitrary two random variables [35]. Let *X* and *Y* represent two random variables respectively; then, the correntropy between them can be defined as

(1) $$V\left(X,Y\right)=E\left(\kappa \left(X,Y\right)\right)=\;\int \int \;\kappa \left(x,y\right)p_{X,Y}\left(x,y\right)dxdy,$$

where E(·) is the expectation function, κ(X,Y) is the kernel function, and $p_{X,Y}\left(x,y\right)$ represents the joint probability density function (PDF) of *X* and *Y*.

The most widely used Gaussian kernel function is defined as

(2) $$G_{\sigma }\left(e\right)=\kappa \left(X,Y\right)=\exp-\left(\frac{{∥X-Y∥}^{2}}{2\sigma^{2}}\right)\;\left(e=X-Y\right),$$

where σ represents the Gaussian kernel bandwidth.

To better capture the heavy-tailed features in the noise, Student’s *t* kernel function [33] is used in this paper to replace the Gaussian kernel function, defined as

(3) $$S_{v,\sigma }\left(e\right)=\kappa_{v,\sigma }\left(X,Y\right)={\left(1+\frac{{∥X-Y∥}^{2}}{v\sigma^{2}}\right)}^{-\left(\frac{v+2}{2}\right)}\;\left(e=X-Y\right),$$

where *v* is used to control the shape of Student’s *t* kernel function, and σ is the kernel bandwidth.

In real applications, it is difficult to obtain the joint PDF of random variables. Therefore, the sample mean estimator of correntropy is often used, as shown in the following equation

(4) $$\hat{V}\left(X,Y\right)=\frac{1}{N}\sum_{i=1}^{N}\kappa \left(e_{i}\right),$$

where $e_{i}=x_{i}-y_{i}$, with $\left(x_{i},y_{i}\right),\;i=1,\ldots ,N$ being *N* samples obtained from $p_{X,Y}\left(x,y\right)$.

### 2.2. Kalman Filter

Consider the following linear stochastic system represented by the state-space model

(5) $$\begin{array}{c} x_{k}=F_{k}x_{k-1}+q_{k-1}\\ y_{k}=H_{k}x_{k}+r_{k}, \end{array}$$

where *k* is the discrete time index, $x_{k}\in R^{n}$ is the state vector, $y_{k}\in R^{m}$ is the measurement vector, $F_{k}$ and $H_{k}$ are known as state transition matrix and the measurement matrix. $q_{k-1}\in R^{n}$ and $r_{k}\in R^{m}$ are the mutually independent process and measurement noise, respectively, and satisfy

(6) $$\begin{array}{c} E\left(q_{k-1}\right)=0,\;E\left(r_{k}\right)=0,\\ E\left(q_{k-1}q_{k-1}^{T}\right)=Q_{k-1},\;E\left(r_{k}r_{k}^{T}\right)=R_{k}. \end{array}$$

In general, the KF includes the following two steps:

1. One-step state prediction: The priori state estimate ${\hat{x}}_{k|k-1}$ and the corresponding error covariance matrix $P_{k|k-1}$ can be given by
  (7) $$\begin{array}{cc} \; & {\hat{x}}_{k|k-1}=F_{k}{\hat{x}}_{k-1|k-1}\\ \; & P_{k|k-1}=F_{k}P_{k-1|k-1}F_{k}^{T}+Q_{k-1}. \end{array}$$
2. Measurement update: The posteriori state estimate ${\hat{x}}_{k|k}$ and the corresponding error covariance matrix $P_{k|k}$ can be given by
  (8) $$\begin{array}{cc} \; & {\hat{x}}_{k|k}={\hat{x}}_{k|k-1}+K_{k}\left(y_{k}-H_{k}{\hat{x}}_{k|k-1}\right)\\ \; & P_{k|k}=\left(I-K_{k}H_{k}\right)P_{k|k-1}{\left(I-K_{k}H_{k}\right)}^{T}+K_{k}R_{k}K_{k}{}^{T}\\ \; & K_{k}=P_{k|k-1}H_{k}{}^{T}{\left(H_{k}P_{k|k-1}H_{k}{}^{T}+R_{k}\right)}^{-1}, \end{array}$$
  where $K_{k}$ is the KF gain matrix.

## 3. Student’s *t* Kernel-Based Maximum Correntropy Kalman Filter

The traditional KF is based on the minimum mean square error (MMSE) criterion and performs well under Gaussian noises. However, when the noises are non-Gaussian or large outliers arise in the measurement, the performance of KF may degrade significantly. Correntropy contains second- and higher order moments of the error and is inherently insensitive to outliers. Therefore, the filters based on the maximum correntropy criterion outperform traditional filters in non-Gaussian noise environments. Meanwhile, these filters are more robust to abnormal measurements. However, the performance of these filters is mainly affected by the kernel function and its parameters. The common Gaussian kernel function may overlook the heavy-tailed properties of heavy-tailed noises, which results in a decrease of the estimation accuracy. To better utilize the heavy-tailed features and improve the estimation accuracy of the system state, Student’s *t* kernel function is used to replace the Gaussian kernel function to model and process the heavy-tailed noise.

For the linear system represented by Equation (5), the following equation can be obtained

(9) $$\left[\begin{array}{c} {\hat{x}}_{k|k-1}\\ y_{k} \end{array}\right]=\left[\begin{array}{c} I_{n\times n}\\ H_{k} \end{array}\right]x_{k}+v_{k},$$

where $v_{k}={\left[-{\left(x_{k}-{\hat{x}}_{k|k-1}\right)}^{T}\;r_{k}^{T}\right]}^{T}$ and the corresponding covariance matrix can be given as

(10) $$E\left[v_{k}v_{k}^{T}\right]=\left[\begin{array}{cc} P_{k|k-1} & 0\\ 0 & R_{k} \end{array}\right]=\left[\begin{array}{cc} B_{p}B_{p}^{T} & 0\\ 0 & B_{r}B_{r}^{T} \end{array}\right],$$

where $B_{p}$ and $B_{r}$ can be obtained from the Cholesky decomposition of $P_{k|k-1}$ and $R_{k}$, respectively.

When Student’s *t* kernel function is employed, the cost function based on the maximum correntropy criterion can be given as

(11) $$J=\frac{1}{n+m}\left[\sum_{i=1}^{n}S_{v,\sigma }\left(e_{x,i}\right)+\sum_{j=1}^{m}S_{v,\sigma }\left(e_{y,j}\right)\right],$$

where $S_{v,\sigma }\left(\cdot \right)$ is Student’s *t* kernel function, and $e_{x,i}$ and $e_{y,j}$ represent the *i*th and *j*th element of $e_{x}$ and $e_{y}$, respectively. The $e_{x}$ and $e_{y}$ are given by

(12) $$\begin{array}{cc} \; & e_{x}=B_{p}^{-1}\left(x_{k}-{\hat{x}}_{k|k-1}\right)\\ \; & e_{y}=B_{r}^{-1}\left(y_{k}-H_{k}x_{k}\right). \end{array}$$

According to the maximum correntropy criterion, to obtain the optimal state estimation ${\hat{x}}_{k|k}$, the following equation should be solved

(13) $$\frac{\partial J}{\partial x_{k}}=0.$$

Substituting Equations (11) and (12) into Equation (13), the following equation can be obtained:

(14) $$\begin{array}{cc} \; & \sum_{i=1}^{n}\frac{v\sigma^{2}}{v\sigma^{2}+e_{x,i}^{2}}S_{v,\sigma }\left(e_{x,i}\right)B_{p,i}^{-T}B_{p,i}^{-1}\left(x_{k}-{\hat{x}}_{k|k-1}\right)\\ \; & \;-\sum_{j=1}^{m}\frac{v\sigma^{2}}{v\sigma^{2}+e_{y,j}^{2}}S_{v,\sigma }\left(e_{y,j}\right)H_{k}^{T}B_{r,j}^{-T}B_{r,j}^{-1}\left(y_{k}-H_{k}x_{k}\right)=0, \end{array}$$

where $B_{p,i}$ and $B_{r,j}$ represent the *i*th and the *j*th row of $B_{p}$ and $B_{r}$, respectively.

The matrix form of Equation (14) can be expressed as

(15) $$\begin{array}{cc} \; & B_{p}^{-T}\Lambda_{x}B_{p}^{-1}\left(x_{k}-{\hat{x}}_{k|k-1}\right)-H_{k}^{T}B_{r}^{-T}\Lambda_{y}B_{r}^{-1}\left(y_{k}-H_{k}x_{k}\right)=0, \end{array}$$

where

(16) $$\begin{array}{cc} \; & \Lambda_{x}=\mathrm{diag}\left(\frac{v\sigma^{2}S_{v,\sigma }\left(e_{x,1}\right)}{v\sigma^{2}+e_{x,1}^{2}},\ldots ,\frac{v\sigma^{2}S_{v,\sigma }\left(e_{x,n}\right)}{v\sigma^{2}+e_{x,n}^{2}}\right)\\ \; & \Lambda_{y}=\mathrm{diag}\left(\frac{v\sigma^{2}S_{v,\sigma }\left(e_{y,1}\right)}{v\sigma^{2}+e_{y,1}^{2}},\ldots ,\frac{v\sigma^{2}S_{v,\sigma }\left(e_{y,m}\right)}{v\sigma^{2}+e_{y,m}^{2}}\right). \end{array}$$

Let

(17) $$\begin{array}{cc} \; & {\tilde{P}}_{k|k-1}=B_{p}\Lambda_{x}^{-1}B_{p}^{T}\\ \; & {\tilde{R}}_{k}=B_{r}\Lambda_{y}^{-1}B_{r}^{T}. \end{array}$$

Then, Equation (15) can be rewritten as

(18) $$\left({\tilde{P}}_{k|k-1}^{-1}+H_{k}^{T}{\tilde{R}}_{k}^{-1}H_{k}\right)x_{k}={\tilde{P}}_{k|k-1}^{-1}{\hat{x}}_{k|k-1}+H_{k}^{T}{\tilde{R}}_{k}^{-1}y_{k}.$$

Add and subtract $H_{k}^{T}{\tilde{R}}_{k}^{-1}H_{k}{\hat{x}}_{k|k-1}$ at the right side of Equation (18), where the following equation can be obtained:

(19) $$\begin{array}{c} \left({\tilde{P}}_{k|k-1}^{-1}+H_{k}^{T}{\tilde{R}}_{k}^{-1}H_{k}\right)x_{k}=\left({\tilde{P}}_{k|k-1}^{-1}+H_{k}^{T}{\tilde{R}}_{k}^{-1}H_{k}\right){\hat{x}}_{k|k-1}+H_{k}^{T}{\tilde{R}}_{k}^{-1}\left(y_{k}-H_{k}{\hat{x}}_{k|k-1}\right). \end{array}$$

Then multiplying ${\left({\tilde{P}}_{k|k-1}^{-1}+H_{k}^{T}{\tilde{R}}_{k}^{-1}H_{k}\right)}^{-1}$ at both sides of Equation (19), we have

(20) $$x_{k}={\hat{x}}_{k|k-1}+{\tilde{K}}_{k}\left(y_{k}-H_{k}{\hat{x}}_{k|k-1}\right),$$

where

(21) $${\tilde{K}}_{k}={\left({\tilde{P}}_{k|k-1}^{-1}+H_{k}^{T}{\tilde{R}}_{k}^{-1}H_{k}\right)}^{-1}H_{k}^{T}{\tilde{R}}_{k}^{-1}={\tilde{P}}_{k|k-1}H_{k}^{T}{\left(H_{k}{\tilde{P}}_{k|k-1}H_{k}^{T}+{\tilde{R}}_{k}\right)}^{-1}.$$

Accordingly, the posteriori error covariance matrix $P_{k|k}$ can be given by

(22) $$P_{k|k}=\left(I-{\tilde{K}}_{k}H_{k}\right)P_{k|k-1}{\left(I-{\tilde{K}}_{k}H_{k}\right)}^{T}+{\tilde{K}}_{k}R_{k}{\tilde{K}}_{k}^{T}.$$

It can be seen from Equations (16) and (17) that ${\tilde{K}}_{k}$ is nonlinear with respect to $x_{k}$. Then the fixed-point iterative method is used to solve Equation (20).

In general, the STKKF algorithm can be given as follows:

1. Initialization: The parameters *v* and σ in the Student’s *t* kernel function are chosen appropriately, and a small number $\varepsilon \in R^{+}$ used as an iterative iteration termination condition is given. The initial state ${\hat{x}}_{0}$ and error covariance matrix ${\hat{P}}_{0}$ are set.
2. State prediction: The one-step state prediction ${\hat{x}}_{k|k-1}$ and the corresponding error covariance matrix $P_{k|k-1}$ are the same as those in KF, which can be obtained by Equation (7).
3. Posterior state estimate:
  (a) Calculate the matrix $B_{p}$ and $B_{r}$ by the Cholesky decomposition of $P_{k|k-1}$ and $R_{k}$, respectively.
  (b) Let ${\hat{x}}_{k}\left(l\right)$ represent the state estimate of the *l*th fixed-point iteration. At the first iteration, ${\hat{x}}_{k}\left(l\right)={\hat{x}}_{k}\left(0\right)={\hat{x}}_{k|k-1}$.
  (c) Calculate the state estimate at the (l+1)th iteration by the following equations
    (23) $$\begin{array}{cc} \; & e_{x}=B_{p}^{-1}\left({\hat{x}}_{k}\left(l\right)-{\hat{x}}_{k|k-1}\right)\\ \; & e_{y}=B_{r}^{-1}\left(y_{k}-H_{k}{\hat{x}}_{k}\left(l\right)\right)\\ \; & \Lambda_{x}=\mathrm{diag}\left(\frac{v\sigma^{2}S_{v,\sigma }\left(e_{x,1}\right)}{v\sigma^{2}+e_{x,1}^{2}},\ldots ,\frac{v\sigma^{2}S_{v,\sigma }\left(e_{x,n}\right)}{v\sigma^{2}+e_{x,n}^{2}}\right)\\ \; & \Lambda_{y}=\mathrm{diag}\left(\frac{v\sigma^{2}S_{v,\sigma }\left(e_{y,1}\right)}{v\sigma^{2}+e_{y,1}^{2}},\ldots ,\frac{v\sigma^{2}S_{v,\sigma }\left(e_{y,m}\right)}{v\sigma^{2}+e_{y,m}^{2}}\right)\\ \; & {\tilde{P}}_{k|k-1}=B_{p}\Lambda_{x}^{-1}B_{p}^{T}\\ \; & {\tilde{R}}_{k}=B_{r}\Lambda_{y}^{-1}B_{r}^{T}\\ \; & {\tilde{K}}_{k}={\tilde{P}}_{k|k-1}H_{k}^{T}{\left(H_{k}{\tilde{P}}_{k|k-1}H_{k}^{T}+{\tilde{R}}_{k}\right)}^{-1}\\ \; & {\hat{x}}_{k}\left(l+1\right)={\hat{x}}_{k|k-1}+{\tilde{K}}_{k}(y_{k}-H_{k}{\hat{x}}_{k|k-1}). \end{array}$$
  (d) Check whether the state estimate in this iteration meets the iteration termination condition by Equation (24). If the termination condition is not met, set l=l+1, return to step (c), and continue the next iteration. Otherwise, set the final state estimate ${\hat{x}}_{k|k}={\hat{x}}_{k}\left(l+1\right)$, and go to step 4.
    (24) $$\frac{∥{\hat{x}}_{k}\left(l+1\right)-{\hat{x}}_{k}\left(l\right)∥}{∥{\hat{x}}_{k}\left(l\right)∥}\leq \varepsilon .$$
4. Posterior error covariance update: calculate the corresponding posteriori error covariance matrix $P_{k|k}$ by Equation (22). Set k=k+1 and return to step 2.

**Theorem** **1.** *If v is fixed, when σ→∞, the STKKF will tend to become the KF.*

**Proof.**  As σ→∞, the matrix $\Lambda_{x}$ and $\Lambda_{y}$ in Equation (16) →I. Accordingly, ${\tilde{P}}_{k|k-1}\to P_{k|k-1}$ and ${\tilde{R}}_{k}\to R_{k}$, which means that STKKF reduces to KF.    □

**Theorem** **2.** *If σ is fixed, when v→∞, the STKKF will tend to become the MCKF with bandwidth σ.*

**Proof.**  As v→∞, the following equation holds:

(25) $$\begin{array}{cc} \lim_{v\to \infty }{\left(1+\frac{{∥X-Y∥}^{2}}{v\sigma^{2}}\right)}^{-\left(\frac{v+2}{2}\right)}\; & \;=\lim_{v\to \infty }{\left[{\left(1+\frac{{∥X-Y∥}^{2}}{v\sigma^{2}}\right)}^{\frac{v\sigma^{2}}{{∥X-Y∥}^{2}}}\right]}^{\frac{{-\left(v+2\right)∥X-Y∥}^{2}}{2v\sigma^{2}}}\\ \; & \;=\exp\left(-\frac{{∥X-Y∥}^{2}}{2\sigma^{2}}\right), \end{array}$$

where the equation $\lim_{x\to \infty }\left(1+\frac{1}{x}\right)=e$ is used. Then Student’s *t* kernel function reduces to the Gaussian kernel function, which means that the STKKF tends to become the MCKF.    □

## 4. Convergence Analysis of STKKF

The fixed-point iteration method is used in the STKKF to update the posterior state estimate. To ensure the iterations converge, the convergence of the STKKF is analyzed in this section. The method used is similar to that of the Ref. [36], where only a sufficient condition is given.

The Equation (15) can be rewritten in the following form:

(26) $$\begin{array}{cc} \; & \left[\begin{array}{cc} I^{T} & H_{k}^{T} \end{array}\right]\left[\begin{array}{cc} B_{p}^{-T} & \\ \; & B_{r}^{-T} \end{array}\right]\left[\begin{array}{cc} \Lambda_{x} & \\ \; & \Lambda_{y} \end{array}\right]\left[\begin{array}{cc} B_{p}^{-1} & \\ \; & B_{r}^{-1} \end{array}\right]\left(\left[\begin{array}{c} I\\ H_{k} \end{array}\right]x_{k}-\left[\begin{array}{c} {\hat{x}}_{k|k-1}\\ y_{k} \end{array}\right]\right)=0. \end{array}$$

Let

(27) $$\begin{array}{ccc} \; & D=\left[\begin{array}{cc} B_{p}^{-1} & \\ \; & B_{r}^{-1} \end{array}\right]\left[\begin{array}{c} {\hat{x}}_{k|k-1}\\ y_{k} \end{array}\right],\; & W=\left[\begin{array}{cc} B_{p}^{-1} & \\ \; & B_{r}^{-1} \end{array}\right]\left[\begin{array}{c} I\\ H_{k} \end{array}\right],\\ \; & \Sigma =\left[\begin{array}{cc} \Lambda_{x} & \\ \; & \Lambda_{y} \end{array}\right],\; & e=\left[\begin{array}{c} -e_{x}\\ e_{y} \end{array}\right]=D-Wx_{k}. \end{array}$$

Equation (26) can be rewritten in the following form:

(28) $$W^{T}\Sigma Wx_{k}-W^{T}\Sigma D=0.$$

Then, $x_{k}$ can be given as

(29) $$x_{k}={\left(W^{T}\Sigma W\right)}^{-1}W^{T}\Sigma D.$$

Firstly, function $f\left(x_{k}\right)=x_{k}$ is constructed, and by substituting Equation (16) into Equation (29), $f\left(x_{k}\right)$ can be expressed as

(30) $$\begin{array}{c} \begin{array}{cc} f\left(x_{k}\right) & ={\left(\sum_{i=1}^{m+n}\frac{v\sigma^{2}S_{v,\sigma }\left(e_{i}\right)}{v\sigma^{2}+e_{i}^{2}}W_{i}^{T}W_{i}\right)}^{-1}\left(\sum_{i=1}^{m+n}\frac{v\sigma^{2}S_{v,\sigma }\left(e_{i}\right)}{v\sigma^{2}+e_{i}^{2}}D_{i}W_{i}^{T}\right)=N_{WW}^{-1}N_{WD}, \end{array} \end{array}$$

where $W_{i}$ represents the *i*th row of matrix W, $D_{i}$ is the *i*th component of vector D, and

(31) $$\begin{array}{cc} \; & N_{WW}=\sum_{i=1}^{m+n}\frac{v\sigma^{2}S_{v,\sigma }\left(e_{i}\right)}{v\sigma^{2}+e_{i}^{2}}W_{i}^{T}W_{i},\;\;\;\;\;N_{WD}=\sum_{i=1}^{m+n}\frac{v\sigma^{2}S_{v,\sigma }\left(e_{i}\right)}{v\sigma^{2}+e_{i}^{2}}D_{i}W_{i}^{T}. \end{array}$$

The Jacobian matrix of $f\left(x_{k}\right)$ with respect to $x_{k}$, $\nabla_{x_{k}}f\left(x_{k}\right)$, can be expressed as

(32) $$\nabla_{x_{k}}f\left(x_{k}\right)=\left[\begin{array}{ccc} \frac{\partial }{\partial x_{k,1}}f\left(x_{k}\right) & \cdots & \frac{\partial }{\partial x_{k,n+m}}f\left(x_{k}\right) \end{array}\right],$$

where

(33) $$\begin{array}{cc} \frac{\partial }{\partial x_{k,j}}f\left(x_{k}\right)\; & =\frac{\partial }{\partial x_{k,j}}N_{WW}^{-1}N_{WD}\\ \; & =N_{WW}^{-1}\left(\frac{\partial }{\partial x_{k,j}}N_{WD}\right)N_{WW}^{-1}N_{WD}+N_{WW}^{-1}\left(\frac{\partial }{\partial x_{k,j}}N_{WD}\right)\\ \; & =N_{WW}^{-1}\left(\frac{\partial }{\partial x_{k,j}}N_{WW}\right)f\left(x_{k}\right)+N_{WW}^{-1}\left(\frac{\partial }{\partial x_{k,j}}N_{WD}\right)\\ \; & =N_{WW}^{-1}\left(\sum_{i=1}^{m+n}\frac{v+4}{v\sigma^{2}}e_{i}W_{i}^{j}{\left(1+\frac{e_{i}^{2}}{v\sigma^{2}}\right)}^{-\frac{v+6}{2}}W_{i}^{T}W_{i}\right)f\left(x_{k}\right)\\ \; & \;+N_{WW}^{-1}\left(\sum_{i=1}^{m+n}\frac{v+4}{v\sigma^{2}}e_{i}W_{i}^{j}{\left(1+\frac{e_{i}^{2}}{v\sigma^{2}}\right)}^{-\frac{v+6}{2}}D_{i}W_{i}^{T}\right). \end{array}$$

Then, the following theorem holds.

**Theorem** **3.** *If parameter v is fixed, β>ξ, and $\sigma >\max(\sigma *,\sigma^{+})$, where σ∗ is the solution of φ(v,σ)=β, $\sigma^{+}$ is the solution of ψ(v,σ)=α(0<α<1), then for $\forall x_{k}\in \left\{R^{n}:{∥x_{k}∥}_{1}<\beta \right\}$, Equation (34) holds. The expression of ξ, φ(v,σ), and ψ(v,σ) are shown in Equations (35), (36) and (37), respectively.*

(34) $$\begin{array}{cc} {∥f\left(x_{k}\right)∥}_{1}\leq \beta & \left(\beta >\xi \right)\\ {∥\frac{\partial }{\partial x_{k}}f\left(x_{k}\right)∥}_{1}\leq \alpha & \left(0<\alpha <1\right) \end{array}$$

(35) $$\xi =\frac{\sqrt{n}\sum_{i=1}^{m+n}\left|D_{i}\right|{∥W_{i}^{T}∥}_{1}}{\lambda_{\min}\left(\sum_{i=1}^{m+n}W_{i}^{T}W_{i}\right)}$$

(36) $$\begin{array}{c} \varphi \left(v,\sigma \right)=\frac{\sqrt{n}\sum_{i=1}^{m+n}\left|D_{i}\right|{∥W_{i}^{T}∥}_{1}}{\lambda_{\min}\left(\sum_{i=1}^{m+n}{\left(1+\frac{{\left(\left|D_{i}\right|+\beta {∥W_{i}∥}_{1}\right)}^{2}}{v\sigma^{2}}\right)}^{-\left(\frac{v+4}{2}\right)}W_{i}^{T}W_{i}\right)} \end{array}$$

(37) $$\begin{array}{c} \psi \left(v,\sigma \right)=\frac{\begin{array}{c} \left(v+4\right)\sqrt{n}\sum_{i=1}^{m+n}\left[\left(\left|D_{i}\right|+\beta {∥W_{i}∥}_{1}\right){∥W_{i}∥}_{1}\left(\beta {∥W_{i}^{T}W_{i}∥}_{1}+{∥D_{i}W_{i}^{T}∥}_{1}\right)\right] \end{array}}{v\sigma^{2}\lambda_{\min}\left(\sum_{i=1}^{m+n}{\left(1+\frac{{\left(\left|D_{i}\right|+\beta {∥W_{i}∥}_{1}\right)}^{2}}{v\sigma^{2}}\right)}^{-\left(\frac{v+4}{2}\right)}W_{i}^{T}W_{i}\right)} \end{array}$$

**Proof.**  (38) $${∥f\left(x_{k}\right)∥}_{1}={∥N_{WW}^{-1}N_{WD}∥}_{1}\leq^{\left(a\right)}{∥N_{WW}^{-1}∥}_{1}{∥N_{WD}∥}_{1},$$

where $\left||\cdot \right|{|}_{p}$ is the $l_{p}$-norm of a vector or induced matrix norm defined by ${∥A∥}_{p}=\max_{{∥x∥}_{p}\neq 0}\frac{{∥Ax∥}_{p}}{{∥x∥}_{p}}$, and $\left(a\right)$ comes from the compatibility of the matrix norm and vector norm.

According to the matrix theory, the following equation holds:

(39) $${∥N_{WW}^{-1}∥}_{1}\leq \sqrt{n}{∥N_{WW}^{-1}∥}_{2}=\sqrt{n}\lambda_{\max}\left(N_{WW}^{-1}\right),$$

where $\lambda_{\max}\left(\cdot \right)$ represents the maximum eigenvalue of the matrix.

(40) $$\begin{array}{cc} \lambda_{\max}\left(N_{WWW}^{-1}\right) & \;=\frac{1}{\lambda_{\min}\left(N_{WW}\right)}\\ \; & \;=\frac{1}{\lambda_{\min}\left(\sum_{i=1}^{m+n}\frac{v\sigma^{2}}{v\sigma^{2}+e_{i}^{2}}S_{v,\sigma }\left(e_{i}\right)W_{i}^{T}W_{i}\right)}\\ \; & \;=\frac{1}{\lambda_{\min}\left(\sum_{i=1}^{m+n}{\left(1+\frac{e_{i}^{2}}{v\sigma^{2}}\right)}^{-\left(\frac{v+4}{2}\right)}W_{i}^{T}W_{i}\right)}\\ \; & \;\left.=\frac{1}{\lambda_{\min}\left(\sum_{i=1}^{m+n}{\left(1+\frac{{\left(D_{i}-W_{i}x_{k}\right)}^{2}}{v\sigma^{2}}\right)}^{-\left(\frac{v+4}{2}\right)}W_{i}^{T}W_{i}\right)}\right.\\ \; & \;\leq^{\left(b\right)}\frac{1}{\lambda_{\min}\left(\sum_{i=1}^{m+n}{\left(1+\frac{{\left(\left|D_{i}\right|+\beta {∥W_{i}∥}_{1}\right)}^{2}}{v\sigma^{2}}\right)}^{-\left(\frac{v+4}{2}\right)}W_{i}^{T}W_{i}\right)}, \end{array}$$

where $\left(b\right)$ comes from ${∥x_{k}∥}_{1}<\beta$.

Similarly,

(41) $$\begin{array}{cc} {∥N_{WD}∥}_{1} & ={∥\sum_{i=1}^{m+n}\frac{v\sigma^{2}}{v\sigma^{2}+e_{i}^{2}}S_{v,\sigma }\left(e_{i}\right)D_{i}W_{i}^{T}∥}_{1}\\ \; & ={∥\sum_{i=1}^{m+n}{\left(1+\frac{e_{i}^{2}}{v\sigma^{2}}\right)}^{-\left(\frac{v+4}{2}\right)}D_{i}W_{i}^{T}∥}_{1}\\ \; & \leq^{\left(c\right)}\sum_{i=1}^{m+n}{∥D_{i}W_{i}^{T}∥}_{1}\\ \; & \leq \sum_{i=1}^{m+n}|D_{i}|{∥W_{i}^{T}∥}_{1}, \end{array}$$

where $\left(c\right)$ is because ${\left(1+\frac{e_{i}^{2}}{v\sigma^{2}}\right)}^{-\left(\frac{v+4}{2}\right)}<1$ for $\forall e_{i}$ and the convexity of the $l_{1}$ norm.

According to Equations (38), (39) and (41), the following equation holds:

(42) $$\begin{array}{c} {∥f\left(x_{k}\right)∥}_{1}\leq \varphi \left(v,\sigma \right)=\frac{\sqrt{n}\sum_{i=1}^{m+n}|D_{i}|{∥W_{i}^{T}∥}_{1}}{\lambda_{\min}\left(\sum_{i=1}^{m+n}{\left(1+\frac{{\left(\left|D_{i}\right|+\beta {∥W_{i}∥}_{1}\right)}^{2}}{v\sigma^{2}}\right)}^{-\left(\frac{v+4}{2}\right)}W_{i}^{T}W_{i}\right)}. \end{array}$$

If the parameter *v* is fixed, then φ(v,σ) is the monotonically decreasing function of σ, and then we have

(43) $$\begin{array}{cc} \; & \lim_{\sigma \to 0^{+}}\varphi \left(v,\sigma \right)=\infty \\ \; & \lim_{\sigma \to \infty }\varphi \left(v,\sigma \right)=\frac{\sqrt{n}\sum_{i=1}^{m+n}\left|D_{i}\right|{∥W_{i}^{T}∥}_{1}}{\lambda_{\min}\left(\sum_{i=1}^{m+n}W_{i}^{T}W_{i}\right)}=\xi . \end{array}$$

Therefore, for ∀β>ξ, ∃, the unique $\sigma^{*}\in \left(0,\infty \right)$, s.t. $\varphi (v,\sigma^{*})=\beta$. When $\sigma >\sigma^{*}$, φ(v,σ)≤β, that is,

(44) $${∥f\left(x_{k}\right)∥}_{1}\leq \beta .$$

According to the matrix theory, to prove ${∥\nabla_{x_{k}}f\left(x_{k}\right)∥}_{1}\leq \alpha$, we just need to prove ${∥\frac{\partial }{\partial x_{k,j}}f\left(x_{k}\right)∥}_{1}$≤α for ∀j. The Equation (33) is rewritten here:

(45) $$\begin{array}{cc} \frac{\partial }{\partial x_{k,j}}f\left(x_{k}\right) & =N_{WW}^{-1}\left(\sum_{i=1}^{m+n}\frac{v+4}{v\sigma^{2}}e_{i}W_{i}^{j}{\left(1+\frac{e_{i}^{2}}{v\sigma^{2}}\right)}^{-\frac{v+6}{2}}W_{i}^{T}W_{i}\right)f\left(x_{k}\right)\\ \; & \;+N_{WW}^{-1}\left(\sum_{i=1}^{m+n}\frac{v+4}{v\sigma^{2}}e_{i}W_{i}^{j}{\left(1+\frac{e_{i}^{2}}{v\sigma^{2}}\right)}^{-\frac{v+6}{2}}D_{i}W_{i}^{T}\right). \end{array}$$

The following equation can be derived:

(46) $$\begin{array}{cc} \; & {∥N_{WW}^{-1}\left(\sum_{i=1}^{m+n}\frac{v+4}{v\sigma^{2}}e_{i}W_{i}^{j}{\left(1+\frac{e_{i}^{2}}{v\sigma^{2}}\right)}^{-\frac{v+6}{2}}W_{i}^{T}W_{i}\right)f\left(x_{k}\right)∥}_{1}\\ \; & \;\;\leq \frac{v+4}{v\sigma^{2}}{∥N_{WW}^{-1}∥}_{1}{∥\sum_{i=1}^{m+n}e_{i}W_{i}^{j}{\left(1+\frac{e_{i}^{2}}{v\sigma^{2}}\right)}^{-\frac{v+6}{2}}W_{i}^{T}W_{i}∥}_{1}{∥f\left(x_{k}\right)∥}_{1}\\ \; & \;\;\leq^{\left(d\right)}\frac{(v+4)\beta }{v\sigma^{2}}{∥N_{WW}^{-1}∥}_{1}{∥\sum_{i=1}^{m+n}e_{i}W_{i}^{j}{\left(1+\frac{e_{i}^{2}}{v\sigma^{2}}\right)}^{-\frac{v+6}{2}}W_{i}^{T}W_{i}∥}_{1}\\ \; & \;\;\leq^{\left(e\right)}\frac{(v+4)\beta }{v\sigma^{2}}{∥N_{WW}^{-1}∥}_{1}\left[\sum_{i=1}^{m+n}\left(\left|D_{i}\right|+\beta {∥W_{i}∥}_{1}\right){∥W_{i}∥}_{1}{∥W_{i}^{T}W_{i}∥}_{1}\right], \end{array}$$

where $\left(d\right)$ comes from that when $\sigma >\sigma^{*}$, ${∥f\left(x_{k}\right)∥}_{1}\leq \beta$, $\left(e\right)$ is because of the convexity of the $l_{1}$ norm, and $\left|e_{i}W_{i}^{j}\right|=\left|\left(D_{i}-W_{i}x_{k}\right)W_{i}^{j}\right|\leq \left(\left|D_{i}\right|+\beta {∥W_{i}∥}_{1}\right){∥W_{i}∥}_{1}$.

Similarly,

(47) $$\begin{array}{cc} \; & {∥N_{WW}^{-1}\left(\sum_{i=1}^{m+n}\frac{v+4}{v\sigma^{2}}e_{i}W_{i}^{j}{\left(1+\frac{e_{i}^{2}}{v\sigma^{2}}\right)}^{-\frac{v+6}{2}}D_{i}W_{i}^{T}\right)∥}_{1}\\ \; & \;\;\leq \frac{v+4}{v\sigma^{2}}{∥N_{WW}^{-1}∥}_{1}{∥\sum_{i=1}^{m+n}e_{i}W_{i}^{j}{\left(1+\frac{e_{i}^{2}}{v\sigma^{2}}\right)}^{-\frac{v+6}{2}}D_{i}W_{i}^{T}∥}_{1}\\ \; & \;\;\leq \frac{v+4}{v\sigma^{2}}{∥N_{WW}^{-1}∥}_{1}\left[\sum_{i=1}^{m+n}\left(\left|D_{i}\right|+\beta {∥W_{i}∥}_{1}\right){∥W_{i}∥}_{1}{∥D_{i}W_{i}^{T}∥}_{1}\right]. \end{array}$$

According to Equations (40), (46) and (47), the following equation can be obtained:

(48) $$\begin{array}{cc} {∥\frac{\partial }{\partial x_{k,j}}f\left(x_{k}\right)∥}_{1} & \leq \psi (v,\sigma )\\ \; & =\frac{\begin{array}{c} \left(v+4\right)\sqrt{n}\sum_{i=1}^{m+n}\left[\left(\left|D_{i}\right|+\beta {∥W_{i}∥}_{1}\right){∥W_{i}∥}_{1}\left(\beta {∥W_{i}^{T}W_{i}∥}_{1}+{∥D_{i}W_{i}^{T}∥}_{1}\right)\right] \end{array}}{v\sigma^{2}\lambda_{\min}\left(\sum_{i=1}^{m+n}{\left(1+\frac{{\left(\left|D_{i}\right|+\beta {∥W_{i}∥}_{1}\right)}^{2}}{v\sigma^{2}}\right)}^{-\left(\frac{v+4}{2}\right)}W_{i}^{T}W_{i}\right)}. \end{array}$$

Additionally, if the parameter *v* is fixed, then ψ(v,σ) is the monotonically decreasing function of σ; then, we have

(49) $$\begin{array}{cc} \; & \lim_{\sigma \to 0^{+}}\psi \left(v,\sigma \right)=\infty \\ \; & \lim_{\sigma \to \infty }\psi \left(v,\sigma \right)=0. \end{array}$$

Therefore, for ∀α∈(0,1), ∃ the unique $\sigma^{+}$, s.t. $\varphi (v,\sigma^{+})=\alpha$. When $\sigma >\sigma^{+}$, the following equation holds:

(50) $${∥\frac{\partial }{\partial x_{k,j}}f\left(x_{k}\right)∥}_{1}\leq \alpha .$$

Based on the above derivation, we conclude that when the parameter *v* is fixed, $\sigma >\max(\sigma *,\sigma^{+})$, and $x_{k}\in \left\{R^{n}:{∥x_{k}∥}_{1}<\beta \right\}$, the following equations hold:

(51) $$\begin{array}{cc} {∥f\left(x_{k}\right)∥}_{1}\leq \beta & \left(\beta >\xi \right)\\ {∥\frac{\partial }{\partial x_{k}}f\left(x_{k}\right)∥}_{1}\leq \alpha & (0<\alpha <1). \end{array}$$

The theorem is proved completely.    □

By Theorem 3 and the Banach Fixed-Point Theorem [37], if the $l_{1}$-norm of the initial iteration point ${∥{\hat{x}}_{k}\left(0\right)∥}_{1}\leq \beta$, then STKKF will surely converge to the unique point in range $x_{k}\in \left\{R^{n}:{∥x_{k}∥}_{1}\leq \beta \right\}$, provided that the kernel bandwidth σ is larger than a certain value.

The implementation pseudocode of the STKKF is shown in Algorithm 1.
**Algorithm 1:** The implementation pseudocode for one time-step of the STKKF.**Inputs:**${\hat{x}}_{k-1|k-1}$, $P_{k-1|k-1}$, $Q_{k-1}$, $R_{k}$, *v*, σ, ε.**Time update:**1. ${\hat{x}}_{k|k-1}=F_{k}{\hat{x}}_{k-1|k-1}$.2. $P_{k|k-1}=F_{k}P_{k-1|k-1}F_{k}^{T}+Q_{k-1}$.**Measurement update:**1. $B_{p}=Chol\left(P_{k|k-1}\right),B_{r}=Chol\left(R_{k}\right)$, ${\hat{x}}_{k}\left(l=0\right)={\hat{x}}_{k|k-1}$.2. $e_{x}=B_{p}^{-1}\left({\hat{x}}_{k}\left(l\right)-{\hat{x}}_{k|k-1}\right),e_{y}=B_{r}^{-1}\left(y_{k}-H_{k}{\hat{x}}_{k}\left(l\right)\right)$.3. $\Lambda_{x}=\mathrm{diag}\left(\frac{v\sigma^{2}S_{v,\sigma }\left(e_{x,1}\right)}{v\sigma^{2}+e_{x,1}^{2}},\ldots ,\frac{v\sigma^{2}S_{v,\sigma }\left(e_{x,n}\right)}{v\sigma^{2}+e_{x,n}^{2}}\right)$,    $\Lambda_{y}=\mathrm{diag}\left(\frac{v\sigma^{2}S_{v,\sigma }\left(e_{y,1}\right)}{v\sigma^{2}+e_{y,1}^{2}},\ldots ,\frac{v\sigma^{2}S_{v,\sigma }\left(e_{y,m}\right)}{v\sigma^{2}+e_{y,m}^{2}}\right)$.4. ${\tilde{P}}_{k|k-1}=B_{p}\Lambda_{x}^{-1}B_{p}^{T},{\tilde{R}}_{k}=B_{r}\Lambda_{y}^{-1}B_{r}^{T}$.5. ${\tilde{K}}_{k}={\tilde{P}}_{k|k-1}H_{k}^{T}{\left(H_{k}{\tilde{P}}_{k|k-1}H_{k}^{T}+{\tilde{R}}_{k}\right)}^{-1}$.6. ${\hat{x}}_{k}\left(l+1\right)={\hat{x}}_{k|k-1}+{\tilde{K}}_{k}\left(y_{k}-H_{k}{\hat{x}}_{k|k-1}\right)$.7. Check $\frac{∥{\hat{x}}_{k}\left(l+1\right)-{\hat{x}}_{k}\left(l\right)∥}{∥{\hat{x}}_{k}\left(l\right)∥}\leq \varepsilon$, where if the termination condition is met, then set ${\hat{x}}_{k|k}={\hat{x}}_{k}\left(l+1\right)$ and go to Step 8; otherwise, set l=l+1 and return to Step 2, and continue the next iteration.8. $P_{k|k}=\left(I-{\tilde{K}}_{k}H_{k}\right)P_{k|k-1}{\left(I-{\tilde{K}}_{k}H_{k}\right)}^{T}+{\tilde{K}}_{k}R_{k}{\tilde{K}}_{k}^{T}$.**Outputs:**${\hat{x}}_{k|k}$, $P_{k|k}$.

## 5. Simulations and Results

In this section, simulations are conducted to demonstrate the performance of STKKF. The results of KF, HKF, MCKF, and STKKF are compared when different kinds of noise distribution exist.

The benchmark navigation problem is considered [38]. The dynamics and measurement model are given as follows:

(52) $$\begin{array}{cc} x_{k} & =\left[\begin{array}{cccc} 1 & 0 & \Delta t & 0\\ 0 & 1 & 0 & \Delta t\\ 0 & 0 & 1 & 0\\ 0 & 0 & 0 & 1 \end{array}\right]x_{k-1}+q_{k}\\ y_{k} & =\left[\begin{array}{cccc} 1 & 0 & 0 & 0\\ 0 & 1 & 0 & 0 \end{array}\right]x_{k}+r_{k}, \end{array}$$

where Δt is the sample period, and $q_{k}$ and $r_{k}$ represent the process noise and measurement noise respectively. The first two components of state vector $x_{k}\in R^{4}$ represent the north and east position of a land vehicle, and the last two components are the corresponding north velocity and east velocity. The position of the vehicle is measured directly by a device.

In the simulation, the sample period Δt is 1 s, and the initial values of the true state $x_{0}$, estimated state ${\hat{x}}_{0|0}$, and error covariance matrix $P_{0|0}$ are assumed to be

(53) $$\begin{array}{c} x_{0}=\left[1,1,1,1\right]\\ {\hat{x}}_{0|0}=\left[1,1,1,1\right]+N\left(0,P_{0|0}\right)\\ P_{0|0}=\mathrm{diag}\left(0.1,0.1,0.1,0.1\right). \end{array}$$

Two different cases with different kinds of processes and measurement noises are considered in this simulation as follows:

1. The process noise and measurement noise are both Gaussian noises.
  (54) $$\begin{array}{cc} \; & q_{k}∼N\left(0,\mathrm{diag}\left(0.1^{2},0.1^{2},0.1^{2},0.1^{2}\right)\right)\\ \; & r_{k}∼N\left(0,\mathrm{diag}\left(1,1\right)\right). \end{array}$$
2. The process noise is a Gaussian distribution and the measurement noise is a Gaussian mixture noise.
  (55) $$\begin{array}{cc} \; & q_{k}∼N\left(0,\mathrm{diag}\left(0.1^{2},0.1^{2},0.1^{2},0.1^{2}\right)\right)\\ \; & r_{k}∼0.9N\left(0,\mathrm{diag}\left(1,1\right)\right)+0.1N\left(0,\mathrm{diag}\left(10^{2},10^{2}\right)\right). \end{array}$$

The proposed STKKF and the other filters are coded with MATLAB, and the simulations are run on a computer with Intel Core i7-3540M CPU at 3.0 GHz. The time steps in the simulation is 200. For each case, 100 Monte Carlo simulations are implemented to quantify estimation performance. The performance of the filters is evaluated by the root mean square error (RMSE) and average RMSE (ARMSE). The RMSE and ARMSE in position are defined as:

(56) $$\begin{array}{cc} \; & {\mathrm{RMSE}}_{\mathrm{pos}}\left(i\right)=\sqrt{\frac{1}{M}\sum_{j=1}^{M}\left({\left(x_{i,1}^{j}-{\hat{x}}_{i,1}^{j}\right)}^{2}+{\left(x_{i,2}^{j}-{\hat{x}}_{i,2}^{j}\right)}^{2}\right)}\\ \; & {\mathrm{ARMSE}}_{\mathrm{pos}}=\frac{1}{K}\sum_{i=1}^{K}{\mathrm{RMSE}}_{\mathrm{pos}}\left(i\right), \end{array}$$

where *M* is the number of Monte Carlo simulations and *K* is the time steps in every Monte Carlo simulation. $\left(x_{i,1}^{j},x_{i,2}^{j}\right)$ and $\left({\hat{x}}_{i,1}^{j},{\hat{x}}_{i,2}^{j}\right)$ are the true position and estimated position at the *i*th time step in the *j*th simulation. The RMSE and ARMSE in velocity are similarly defined.

**Figure 1:**
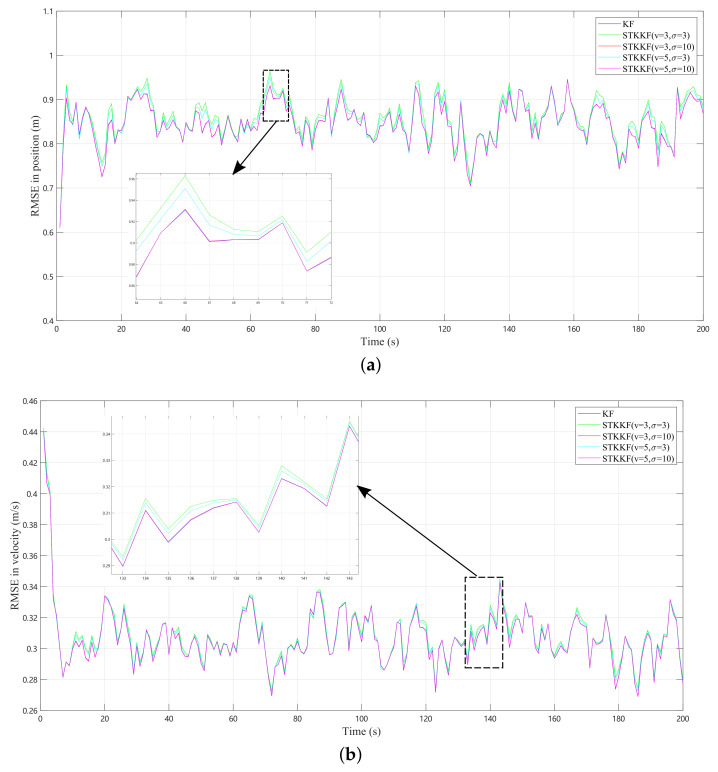
RMSE results of KF and STKKF in case 1 where RMSE is taken over 100 Monte Carlo simulations: (**a**) position. (**b**) velocity.

In case 1, the process and measurement noise are both Gaussian noises. Theoretically, KF should be the best estimator. To demonstrate the relationship between the KF and STKKF, these two filters were studied in this case. The ARMSE of the position and velocity estimates are listed in Table 1. Meanwhile, the average iteration number required for the STKKF to converge and the average implementation times of the filters for one time step are also listed. The iteration termination condition is given by Equation (24). Here, ε is set as $10^{-4}$. In addition, the RMSEs of the position and velocity of KF and STKKF with different kernel parameters are also plotted in Figure 1a and Figure 1b, respectively.

**Figure 2:**
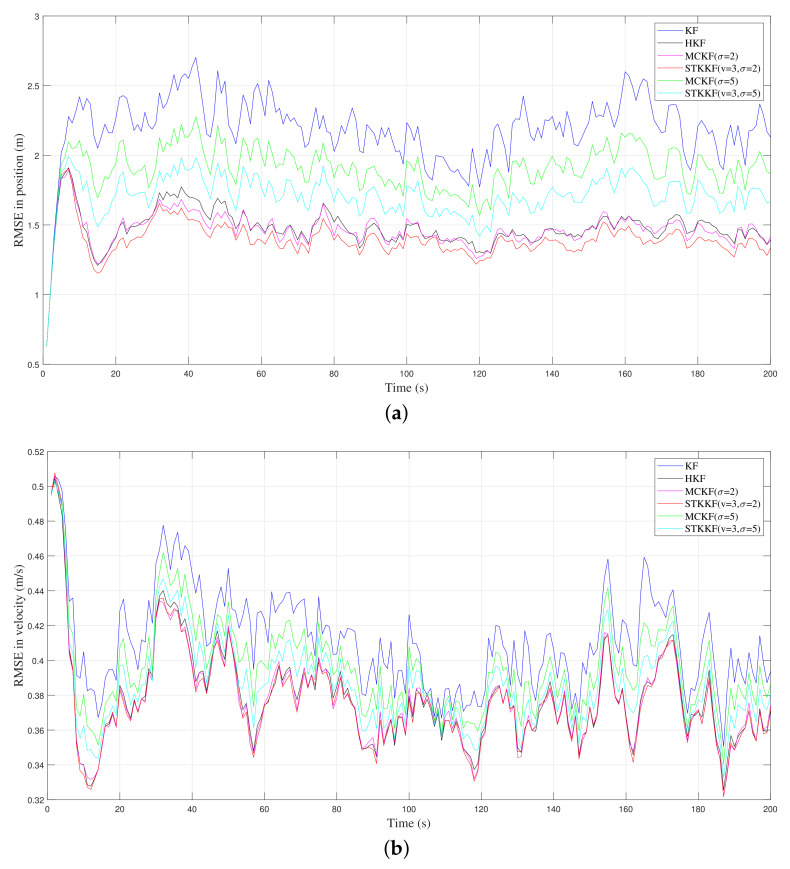
RMSE results of different filters in case 2 where RMSE is taken over 100 Monte Carlo simulations: (**a**) position. (**b**) velocity.

In case 2, the process noises are still Gaussian but the measurement noise is a heavy-tailed (impulsive) non-Gaussian noise, with a mixed-Gaussian distribution. The ARMSEs of the position and velocity estimate of different filters, average iteration numbers for MCKF and STKKF, and the average implementation times of the filters for one time step are listed in Table 2. In MCKF, the iteration termination parameter is set to be the same as that of STKKF, that is, $10^{-4}$. The RMSEs of the position and velocity of different algorithms are also plotted in Figure 2a and Figure 2b, respectively. It should be noted that for the MCKF and STKKF with different kernel parameters listed in Table 2, only partial models of them are plotted in the figures to maintain the clarity of the plot. It should be pointed out that the noise parameters of KF in the Table 2 are set with the true noise covariance of mixture distribution in Equation (55), and hence, KF is the best linear estimator in the MSE sense. The parameter used in the HKF is set to ensure that the best estimation accuracy is obtained.

## 6. Discussion

In this paper, the Student’s *t* kernel function is employed to replace the traditional Gaussian kernel function in the definition of correntropy to better utilize the heavy-tailed features of noises when the underlying system is disturbed by heavy-tailed non-Gaussian noise. Then the maximum correntropy criterion based on Student’s *t* kernel function is applied to the Kalman filter as the optimality criterion. Based on the criterion, a novel Kalman-type filtering algorithm, named STKKF, is derived. Meanwhile, since the fixed-point iteration method is used to update the posterior state estimate, the convergence of the STKKF is also analyzed.

The performance of the proposed filter is verified through comparative simulations with KF, HKF, and MCKF. When both the process noise and measurement noise are Gaussian noises, it can be seen from Table 1 and Figure 1 that the performance of the KF is the best, since both the noises are Gaussian. When the kernel bandwidth is too small, the STKKF may achieve worse performance. However, one can also see that with the increase of the kernel bandwidth, the performance of the STKKF becomes better and approaches that of the KF. This phenomenon can be explained by Theorem 1, that is, when the filter kernel bandwidth σ→∞, the STKKF will tend to become the KF algorithm. In general, with appropriate parameters, the STKKF performance is at least as good as KF. In addition, the average fixed-point iteration numbers required for STKKF to converge and the average implementation times of the filters for one time step are also calculated in Table 1. It is obvious that the average iteration numbers decrease as the kernel bandwidth σ increases, that is, the convergence speed becomes faster. Correspondingly, the average implementation time of STKKF also decreases. In practical real-time applications, the kernel bandwidth should be set appropriately to ensure the algorithm can run in real time.

When the measurement noise is a heavy-tailed (impulsive) non-Gaussian noise, the results shown by Table 2 and Figure 2 demonstrate that with appropriate parameters, the state estimate accuracy of filters based on the maximum correntropy criterion (MCKF and STKKF) outperform that of the other algorithms. However, the implementation times of these maximum correntropy filters are much longer than that of KF and HKF due to their computational complexity. Furthermore, it can be seen that the implementation times of STKKF are consistently longer than MCKF when they have the same kernel bandwidth. This is because the shape of Student’s *t* kernel function has a heavier tail than the Gaussian kernel function. In addition, one can also see that when the kernel bandwidth is set as the same, the performance of the STKKF with different *v* is consistently better than MCKF. Additionally, with the increase of the *v*, the performance of the STKKF approaches that of the MCKF. This phenomenon can be explained by Theorem 2, that is, when v→∞, the STKKF will tend to become the MCKF with bandwidth σ.

In summary, with proper parameters, the STKKF can outperform the other filtering algorithms, especially for the heavy-tailed non-Gaussian noises. However, like other filters based on the maximum correntropy criterion, the choice of parameters of the kernel function is critical. When the parameters are not appropriate, the filter’s performance may degrade, which should be given much attention in practical applications. At present, the Student’s *t* kernel function-based maximum correntropy criterion is only being applied to the linear system. In the future, an extension to the nonlinear system model can be investigated.

## Figures and Tables

**Table 1:** ARMSEs of position and velocity, average iteration numbers, and average implementation times for one time step of the proposed STKKF and KF when both the process noise and measurement noise are Gaussian noises.

Filters	ARMSE${}_{pos}$ (m)	ARMSE${}_{vel}$ (m/s)	Average Iteration Number	Time (ms)
KF	0.8459	0.3037	0	0.0140
STKKF(v=3,σ=3)	0.8585	0.3091	3.5356	0.1386
STKKF(v=3,σ=10)	0.8461	0.3073	2.3422	0.1008
STKKF(v=3,σ=50)	0.8459	0.3073	2.0124	0.0887
STKKF(v=5,σ=3)	0.8528	0.3083	2.3100	0.1314
STKKF(v=5,σ=10)	0.8460	0.3073	2.2833	0.0954
STKKF(v=5,σ=50)	0.8459	0.3073	2.0083	0.0877

**Table 2:** ARMSEs of position and velocity, average iteration numbers, and average implementation times for one time step of different algorithms when the measurement noise is mixed Gaussian noise.

Filters	ARMSE${}_{\mathrm{pos}}$ (m)	ARMSE${}_{\mathrm{vel}}$ (m/s)	Average Iteration Number	Time(ms)
KF	2.1938	0.4118	0	0.0138
HKF	1.4792	0.3784	0	0.0214
MCKF (σ=2)	1.4662	0.3777	2.5260	0.0820
STKKF (v=3,σ=2)	1.3965	0.3768	2.7070	0.1094
STKKF (v=10,σ=2)	1.4314	0.3769	2.5898	0.1031
STKKF (v=50,σ=2)	1.4575	0.3775	2.5390	0.1013
MCKF (σ=3)	1.6357	0.3838	2.4094	0.0797
STKKF (v=3,σ=3)	1.4837	0.3783	2.5420	0.1011
STKKF (v=10,σ=3)	1.5697	0.3812	2.4602	0.0994
STKKF (v=50,σ=3)	1.6201	0.3832	2.4211	0.0946
MCKF (σ=5)	1.9051	0.3963	2.2782	0.0745
STKKF (v=3,σ=5)	1.7110	0.3871	2.3907	0.0980
STKKF (v=10,σ=5)	1.8318	0.3927	2.3236	0.0964
STKKF (v=50,σ=5)	1.8890	0.3955	2.2883	0.0937

## Data Availability

The data presented in this study are available on request from the corresponding author.

## Abbreviations

The following abbreviations are used in this manuscript:

KF	Kalman filter
EKF	Inertial Measurement Unit
UKF	Unscented Kalman filter
QKF	Quadrature Kalman filter
CKF	Cubature Kalman filter
HKF	Huber-based Kalman filter
MCKF	Maximum correntropy Kalman filter
MEEKF	Minimum error entropy Kalman filter
CKKF	Cauchy kernel-base maximum correntropy Kalman filter
STKKF	Student’s t kernel-based maximum correntropy Kalman filter
MCC	Maximum correntropy criterion
UT	Unscented transformation
MSE	Mean square error
PDF	Probability density function
